# Successful Treatment of Gastric Relapse in Multiple Myeloma with Bortezomib after Autologous Hematopoietic Stem Cell Transplantation (autoHSCT)

**DOI:** 10.4084/MJHID.2013.006

**Published:** 2013-01-02

**Authors:** Serdar Sivgin, Suleyman Baldane, Leylagul Kaynar, Fatih Kurnaz, Mevlut Baskol, Mustafa Kula, Celalettin Eroglu, Kemal Deniz, Bulent Eser, Ali Unal, Mustafa Cetin

**Affiliations:** 1 Dedeman Stem Cell Transplantation Hospital, Department of Hematology, Faculty of Medicine, Erciyes University, Kayseri, Turkey; 2 Department of Internal Medicine, Faculty of Medicine, Erciyes University, Kayseri, Turkey; 3 Department of Gastroenterology, Faculty of Medicine, Erciyes University, Kayseri, Turkey; 4 Department of Nuclear Medicine, Faculty of Medicine, Erciyes University, Kayseri, Turkey; 5 Department of Radiation Oncology, Faculty of Medicine, Erciyes University, Kayseri, Turkey; 6 Department of Pathology, Faculty of Medicine, Erciyes University, Kayseri, Turkey.

## Abstract

We report a case of 59-year-old Turkish man with history of mitral valve replacement (MVR) and chronic obstructive pulmonary disease (COPD) who was diagnosed with stage IIIA IgG lambda multiple myeloma (MM) in 1997. He underwent autologous hematopoietic stem cell transplantation after a conditioning regimen with melphalan 200mg per body area (m^2^) in February 2006. On February 2011, he was admitted to the emergency service of university hospital with complaints of hematemesis and melena. Pathological evaluation of gastric biopsy, obtained from a lesion of small gastric curvature, showed the gastric mucosa infiltrated by neoplastic plasma cells, monoclonal lambda light chain positive. The patient was considered as having local gastric relapsed disease and was treated with 2 cycles of bortezomib. He achieved an excellent local response after 2 cycles of bortezomib, cyclophosphamide and prednisone (BEP) regimen, with healing of gastric ulcer and no recurrence of the hematemesis or melena.

## Introduction

Extramedullary accumulation of plasma cells, defined plasmocytoma, occur in up to 20% of patients with multiple myeloma.^1^–^3^ The most common site for extramedullary involvement is the upper aero digestive tract, which includes the oronasal pharynx, nasal cavities, sinuses and larynx.^4^–^6^ Plasma cell infiltration can involve also any segment of the gastro intestinal tract, representing only 5% of patients with extramedullary involvement.^7^ The most common site involved in the gastrointestinal tract is the small bowel, the involvement of this region presents intestinal obstruction and malabsorption.^3^,^4^ Other gastrointestinal sites are the stomach, colon and oesophagus, in order of frequency of involvement.^8^,^9^ The involvement of the gastrointestinal tract years after an initial diagnosis of MM is exceptional and, when reported, always associated with a poor prognosis.^10^,^11^ We report a case of 59-year-old man with MM with gastric relapse who had been treated successfully with bortezomib and achieved remission after autologous hematopoietic stem cell transplantation (autoHSCT).

## Case Presentation

**Figure 1:**
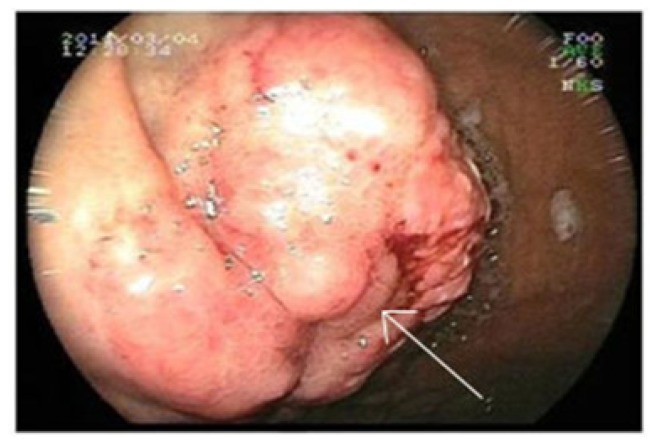
Pre-treatment endoscopic examination shows a large bleeding mass in the small curvature of the stomach

We report a case of 59-year-old man with history of MVR and COPD diagnosed with IgG lambda MM (stage IIIA) in 1997. He was initially treated with six cycles of vincristine, doxorubicin and dexamethasone, followed by high-dose melphalan (10mg/m^2^, 1–4 days, orally) and cyclophosphamide (50mg/day, continuously, orally) which resulted in a complete response with a bone marrow plasma cell lower than <5% for 12 months. He received maintenance therapy with melphalan (10mg/m^2^) for 2 years. In July 2005; he was admitted to emergency service with complaints of bone ache and fatigue. The patient relapsed with rapidly progressing disease, characterized by an increased paraprotein level (15 g/l), a mildly raised LDH level (295 U/l). In bone marrow aspiration analysis; plasma cell ratio was found > 30% and considered as relapsed status. He was treated with vincristine, doxorubicin and dexamethasone (VAD) for 4 cycles. After achieving complete remission (no finding of the original monoclonal paraprotein in serum and urine, also in bone marrow aspirate plasma cells were found <5%) he underwent autologous hematopoietic stem cell transplantation following melphalan 200mg per body area (m^2^) in Stem Cell Transplantation Hospital, Department of Hematology, Erciyes University, Kayseri, Turkey in February 2006. The patient was followed up in complete remission in outpatient clinic of the hospital with regular control sessions. On February 2011; he was admitted to the emergency service of university hospital with complaints of hematemesis and melena. The patient has been using warfarin for prophylaxis of thromboembolism after MVR operation. The laboratory parameters were as follows; prothrombin time (PT): 26,8 sec (10.1–14.9), activated partial thromboplastin time (aPTT): 31,3 sec (25–35), INR:2,48 (0.8–1.2) in total blood count, white blood cell (WBC):6,11 × 10^3^/μL, hemoglobin(Hb): 7,7 g/dL (14.0–18.0) and platelet count (PLT):169 × 10^3^/μL (130–400) The biochemical tests were; blood urea nitrogen (BUN):29 mg/dL (9–23), creatinin:0,85 mg/dL (0.7–1.23), potassium (K): 4,6 mg/dL (3.5–5.5), calcium (Ca): 8,1 mg/dL (8.3–10.6), LDH:284 U/L (120–246), AST: 19 U/L (0–34), ALT: 9 U/L (10–49), ALP: 64 U/L (45–129), GGT: 101U/L (0–73). The upper gastrointestinal endoscopic examination revealed a bleeding ulcerative lesion with a diameter of 2 cm in the small curvature of the stomach (Figure 1).

**Figure 2:**
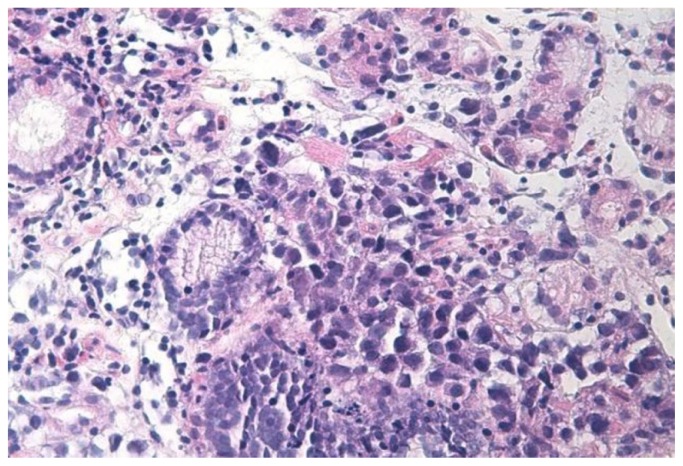
Image of gastric glands in lamina propria infiltrated by plasma cells (H&E section; 400× magnification)

The patient was recommended not taking food and drinks, and He was submitted to gastric decompression with a nasogastric tube. The bleeding was controlled with argon plasma coagulation and sclerotherapy. Biopsy of the gastric lesion showed neoplastic plasma cells, monoclonal lambda light chain positive, infiltrating gastric mucosa (Figure 2).

**Figure 3:**
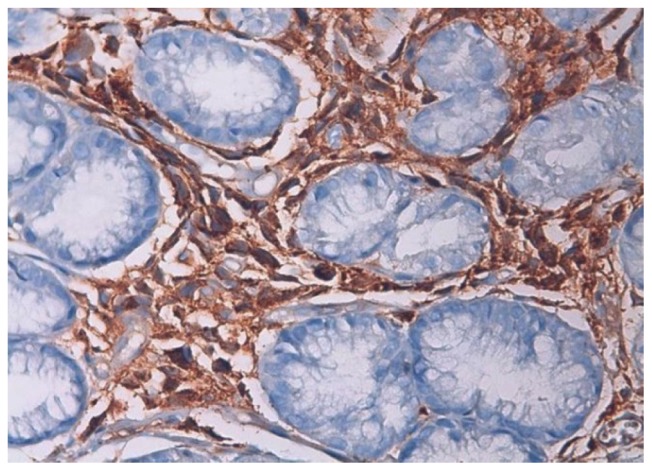
Lambda positive staining of gastric glands in lamina propria (H&E section; 400× magnification)

**Figure 4:**
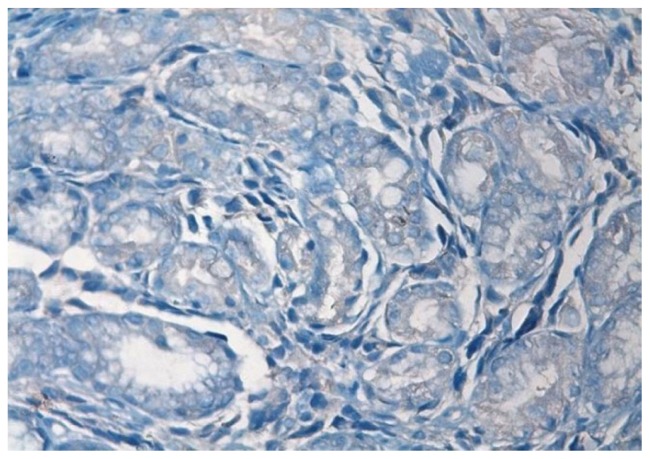
Kappa negative staining of gastric glands in lamina propria (H&E section; 400× magnification)

In immunohistochemistry; staining with CD38 and lambda were positive and kappa was negative. (Figures 3 and 4).

In laboratory tests; serum Ig G was; 1520 mg/dL (650–1600), IgM: 112 mg/dL (50–301), IgA: 342 mg/dL 845–380), kappa: 338 mg/dL (629–1350), lambda: 540 mg/dL (313–723), β2 microglobulin: 2,66 mg/dL (1.16–2.52). A bone marrow biopsy was performed and plasma cell was found below 5 %.

**Figure 5:**
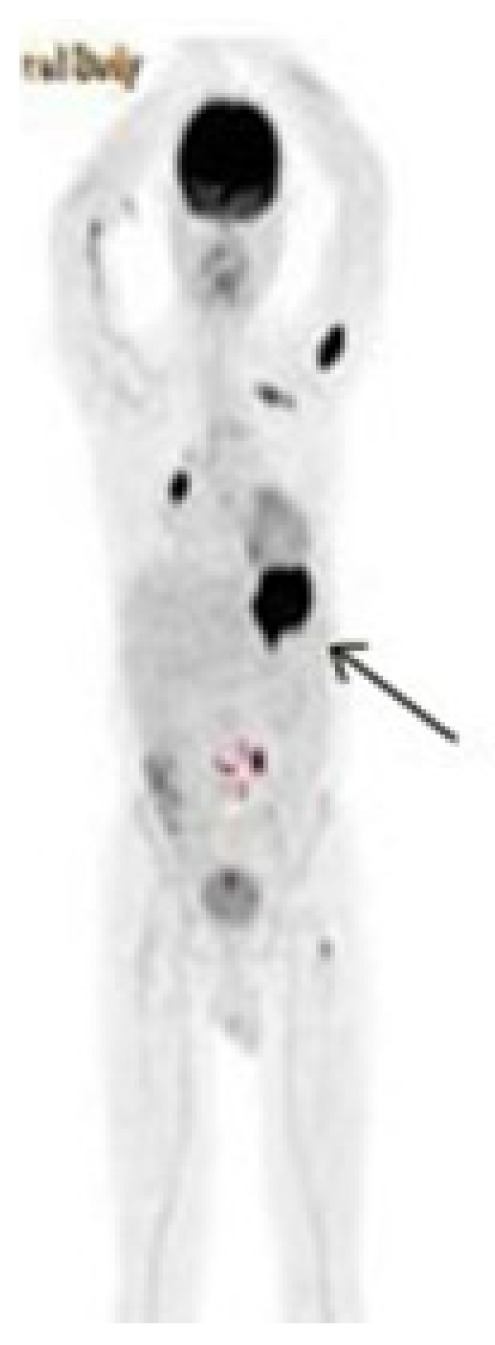
Pre-treatment PET-CT scan of the patient. PET images show presence of intense FDG uptake (SUV_max_ 26.5) in the gastric curvatures with thickening

A PET-CT scan was performed to determine the gastric involvement of the disease. The scan showed dense FDG uptake (suv max: 26,5) in gastric fundus and corpus with a wall thickness of 35 mm (Figure 5).

**Figure 6:**
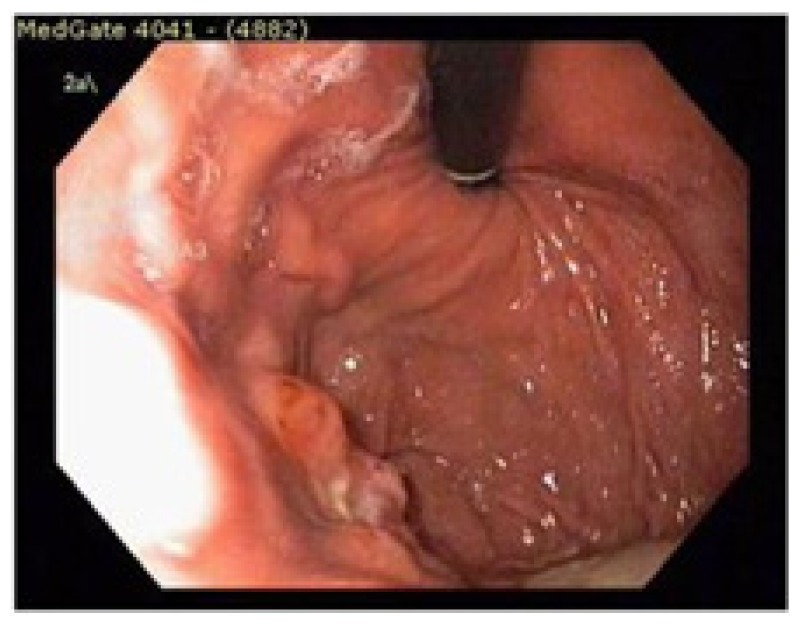
Post-treatment endoscopic screening shows no residual tumor mass

Serum immunofixation test showed no monoclonality and total blood count was in normal ranges. Through these findings, the patient was considered as local gastric relapsed disease and was treated with 2 cycles of bortezomib at a dose of bortezomib 1,3 mg/m^2^, on days 1,4,8 and 11 intravenously, oral cyclophosphamide 50 mg per day continously and oral prednisone 100 mg per day on days 1,4,8 and 11. The cycle was repeated every 3 weeks. After the chemotherapy, control upper gatrointestinal endoscopy was performed and found that the lesion was completely resolved. (Figure 6).

**Figure 7:**
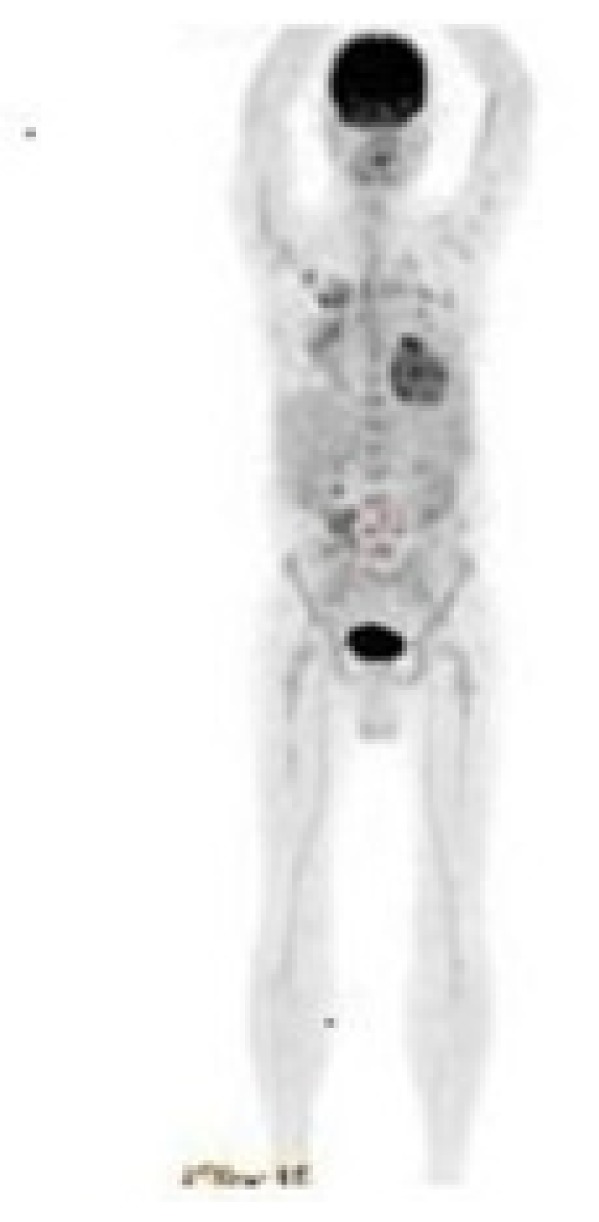
Post-treatment PET-CT scan showing no residual mass in the abdomen

**Figures 8 and 9:**
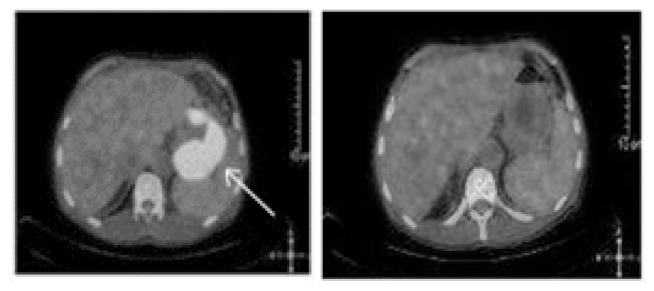
Pre and post-treatment PET fusion images of the patient. The dramatic shrinkage of the gastric mass is exactly shown between the images

Also, we performed a PET-CT scan to determine the last status of the lesions in the body. PET-CT scan showed no residual lesion in whole body and. showed a dramatic shrinkage of the gastric mass (Figure 7) also shown in PET-CT fusion images (Figures 8 and 9).

An excellent response was achieved after 2 cycles of BEP regimen, the paraprotein level was not detectable and there was no recurrence of the hematemesis or melena. His general condition improved rapidly and he was discharged after the second cycle had commenced.

## Discussion

Gastrointestinal involvement in MM is very rare. It most often occurs in the context of an isolated, primary, extramedullary plasmacytoma.^12^ Patients with newly diagnosed MM rarely present with symptoms which are related to gastrointestinal involvement.^13^

Multiple myeloma is a clonal malignancy of plasma cells characterized by the development of anemia. The malignant plasma cells are usually confined to the bone marrow, relying on the marrow stroma for their growth and survival.^14^ Extramedullary involvement is rare, accounting for 14% of relapses following autologous stem-cell transplantation, with fewer than 5% of those with extramedullary disease having gastrointestinal involvement.^7^ In our patient both gastric biopsy and PET-CT scan showed gastric involvement of multiple myeloma admitted with upper gastrointestinal bleeding. Benusiglio et al reported a patient with MM whom had gastrointestinal relapse.^15^ The patient was successfully treated with lenalidomide and achieved a long-lasting response to treatment. The clinican should take into account gastric involvement in patients with MM presenting gastrointestinal bleeding. In our case, the gastric involvement -which can be considered as a rare event- was successfully treated with bortezomib. Our data suggest that features and treatment modalities of extramedullary plamocytoma can be different and that the extramedullary localization is most frequent in genomically defined high-risk multiple myeloma; however, in any case, extramedullary disease is associated with shorter progression-free survival and overall survival.^16^–^20^

The prognosis following an extra medullary relapse of myeloma is generally significantly worse than for medullary relapse, with most patients having few remaining therapeutic options. Some groups, however, have demonstrated that an individualized treatment Schedule following extramedullary relapse could be successful in controlling the disease and could offer survival rates that are comparable to those seen following medullary relapse.^21^ In our patient, prognosis was excellent after the treatment with bortezomib and, at present, the patient is under routine control. Traditionally, nuclear medicine scans exploiting the increased metabolic activity of hematological tumors, such as lymphoma, have not been used in the staging and monitoring of myeloma patients; however, recent evidence supports the use of modalities such as FDG-PET for the early diagnosis of relapsed myeloma. The recent data suggests that this technique is especially useful in the diagnosis and monitoring of aggressive extramedullary disease.^22^ The data showed that 18F-FDG PET/CT could be used for staging, identifying optimal sites for biopsy, restaging, and monitoring response to treatment for MM and related plasma cell dyscrasias.^23^ Gozzetti et al^24^ stated that imaging techniques have long been used to help diagnose patients and determine the stage of the disease, especially PET-CT. In a recent study,^25^ it the importance and feasibility of imaging techniques like PET-CT, have been demonstrated. This study showed that PET-CT has an important role on diagnosis and staging of solitary plasmacytoma, also determining the response to treatment. In our case, PET-CT scan was used to determine involvemnet of all body sites including gastric mucosa and also after chemotherapy to assess the effect of the treatment. 18F-FDG uptake in the stomach could be considered as a non-specific finding and physicians should differentiate several benign disorders.

## Conclusion

In this case, we experienced that bortezomib was very effective in patient with gastric involvement. It reminds us that, in addition to much more common causes (for example, ulcers), the clinician must consider gastrointestinal involvement in patients with MM presenting gastrointestinal hemorrhage. It also shows that patients with MM who have been heavily pre-treated can benefit from novel drugs, like bortezomibeven when they are critically ill. Finally, we should emphasized that a response to this drug was obtained despite active bleeding in the upper gastrointestinal system.
